# Factors associated with health decision-making autonomy on own healthcare among Tanzanian women: A 2022–2023 demographic health survey study

**DOI:** 10.1371/journal.pone.0302191

**Published:** 2025-05-23

**Authors:** Tausi Seleman Haruna, Henry Ofori Duah, Rebecca Lee

**Affiliations:** 1 Fundamentals of Nursing and Basic Sciences, Hubert Kairuki Memorial University, Dar es Salaam, Tanzania; 2 College of Nursing, University of Cincinnati, Cincinnati, Ohio, United States of America; PLOS: Public Library of Science, UNITED KINGDOM OF GREAT BRITAIN AND NORTHERN IRELAND

## Abstract

**Background:**

Women’s health decision-making autonomy is fundamental for the health and well-being of women and their children. It empowers women to make health decisions and exercise their rights and choices surrounding their health. Like most parts of Africa, women’s autonomy in Tanzania remains contentious, with an estimated 19% prevalence of health decision-making autonomy in 2015. Given the impact of women’s health decision-making autonomy on women’s health outcomes and the fact that women’s health decision-making autonomy is an ongoing process affected by advancements in technology, economic growth, and social and cultural shifts, understanding the sociodemographic correlates of women’s autonomy is imperative.

**Objective:**

To examine the factors associated with health decision-making autonomy on their own health among Tanzanian women aged 15–49.

**Methods:**

A non-experimental cross-sectional study using secondary data from the current Tanzania Demographic and Health Survey and Malaria Indicator Survey (TDHS-MIS) 2022–2023. The R statistical programming language was used to run the analysis. Chi-square and Ordinal Logistic Regression were fitted to identify the sociodemographic characteristics associated with women’s health decision-making autonomy on their own health. The odds ratio with its 95% confidence interval was used to determine the significance level at p-value <0.05. All estimates were adjusted for sample design (sample weight, strata, and sampling units).

**Results:**

A total of 9,249 women were included in the analysis. A large proportion (20%) of women aged 25–29. Only 1,908 (21%) of women had complete autonomy, 4,933 (53%) had joint autonomy, and 2,408 (26%) had no autonomy. Women aged 40–44 years (AOR = 2.15; 95% CI: 1.70, 2.71), a higher education level (AOR = 2.07; 95% CI: 1.39, 3.08), richest household wealth index (AOR = 1.80; 95% CI: 1.39, 2.33), currently working (AOR = 1.61; 95% CI: 1.43, 1.83), and living in the Southwest Highlands zone (AOR = 5.86; 95% CI: 4.47, 7.67) were independently associated with higher odds of complete autonomy in their own healthcare as opposed to no autonomy. Rural residence (AOR = 0.59; 95% CI: 0.46, 0.75) was associated with decreased odds of complete autonomy compared to no autonomy.

**Conclusion:**

These results show that health decision-making autonomy among Tanzanian women remains very low. Efforts to empower women through better education and means to improve their economic status are needed to increase complete health decision-making autonomy on their health.

**Recommendation:**

Accelerated and concerted efforts to increase health decision-making autonomy among married women will eventually improve their health and well-being and that of society.

**Future implications to practice, policy, and research:**

The findings can serve as a basis for exploratory qualitative research to further understand the process of health decision-making autonomy among Tanzanian women. Stakeholders can create focused interventions to improve women’s health decision autonomy, emphasizing education and initiatives that generate income, especially in rural regions. Policymakers are encouraged to continue creating policies that promote women’s education and economic empowerment, as these factors are linked to increased autonomy in healthcare decisions.

## Introduction

Women’s health decision-making autonomy is fundamental for the health and well-being of women and their children. It facilitates access to knowledge, power, and socio-cultural resources within the family and community [1–4]. It empowers women to control their health decisions and exercise their rights and choices regarding their health [5]. Women’s autonomy in healthcare decision-making is one of the indicators of women’s health outcomes and empowerment [2,6]. A unit increase in women’s autonomy scale increases the odds of seeking healthcare by 61% (OR=1.61, 95% *CI* [1.52, 1.69], *p* = 0.000) [6]. The Sustainable Development Goals (SDGs) set a target to reduce maternal mortality to less than 70 deaths per 100,000 live births by 2030 [7]. Without paying attention to women’s health decision-making autonomy, efforts to achieve this goal and improve women’s health outcomes may be compromised [8].

Women’s autonomy is a multifaceted concept comprising cultural, social structures, and economic dimensions [9]. Because of its multidimensional nature, there is no single acceptable definition of women’s autonomy. Studies on women’s autonomy defined it according to context, situation, and purpose [10,11]. Dyson and Moore (1983) referred to women’s autonomy as the technical, social, and psychological ability to obtain information and use it as the basis for making decisions about one’s private concerns and those of one’s intimates. Basu (1992) defined women’s autonomy as the capacity and freedom to act independently, for instance, the ability to go places, such as health facilities or markets, or to make decisions regarding contraceptive use or household purchases alone and without asking anyone’s permission. Mason (1986) defined it as women’s ability to make and execute independent decisions about personal matters of importance to their lives and families. With regard to the healthcare context, women’s autonomy in this study is defined as the ability of women to make independent decisions in their own healthcare [12].

Evidence suggests that healthier and empowered women are better positioned for multiple roles, including contributing to their families’ well-being, participating in the workforce, and engaging in community leadership [13,14]. Further, research indicates that women with greater autonomy are more likely to seek healthcare for themselves and utilize different forms of maternal healthcare services for themselves and their children [6,10,15–17]. However, on the other side, the lack of women’s decision-making autonomy in seeking maternal healthcare has been shown to negatively impact maternal health outcomes [2,17,18]. Literature reports delays in decision-making, which is explained in terms of a lack of women’s autonomy, indirectly contribute to maternal mortality [18–20]. Additionally, poor utilization of maternal services due to limited decision-making power has been reported to result in inadequate prenatal and postnatal care [17]. Reports also show the connection between maternal morbidity and mortality and women’s lack of autonomy in health decision-making [17,21]. This is because women cannot make well-timed and informed decisions about their health [17].

Existing studies in low and middle-income countries (LMICs) show a close correlation between women’s socio-demographic characteristics and health decision-making autonomy [4,10,15,22–27]. A study among Nigerian women examining the perceived health decision-making autonomy about their own healthcare found that geographical region, rural/urban residence, age, education, religion, wealth index, and current working independently associated with women’s health decision-making autonomy [27]. The authors found that the region of residence was the strongest independent factor associated with women’s autonomy in health decisions, even after controlling for other variables [27]. Nigatu et al. (2014) reported that women with a higher wealth index are more likely to have choices regarding their own healthcare compared to their counterparts. In Tanzania, a study by Masawe et al. (2019) found that women with secondary and post-secondary education, older and employed, tend to be more autonomous. Similar findings were reported in Nepal, where women with increased education, employment, and a higher number of living children were associated with increased autonomy in decision-making [22].

Most studies that have examined the association between women’s autonomy and its related factors are reported in Asian countries, with Ethiopia accounting for the majority of studies conducted in Africa [11,28]. According to Osamor & Grady’s (2016) review, there is little data in East African countries about the factors that influence women’s autonomy in making healthcare decisions for themselves. In Tanzania, not enough studies have examined the association between women’s health decision-making autonomy on their own health and its associated factors. Masawe et al. (2019) examined the factors related to decision-making autonomy on one’s health; however, this study did not assess the influence of various country zones on women’s health decision-making autonomy.

Like most parts of Africa, women’s status in Tanzania remains challenging, with an estimated 19% prevalence of health decision-making autonomy in 2015 [26]. This signifies that women have low autonomy in health decision-making. Decisions on maternal healthcare are often made with their husbands/partners or someone else, which negatively influences maternal and child healthcare utilization [29–31]. Government, non-government, and international efforts that promote women’s autonomy in health decision-making have been employed [32]. The Tanzanian government has been striving to implement policies that promote equality and women’s rights and encourage the participation of women in decision-making [33]. The government also enforces strategies that promote access to education for girls and women and enhance women’s participation in higher education and economic empowerment by focusing on programs that seek to improve women’s financial independence [32].

Considering all these efforts and given that women’s health decision-making autonomy is an ongoing process affected by technological and economic growth and social and cultural changes, understanding the sociodemographic correlates of women’s autonomy is imperative. Therefore, this study examined the relationship between health decision-making autonomy and sociodemographic characteristics among Tanzanian women of reproductive age 15–49. This relationship becomes particularly important as various programs in low-income countries, including Tanzania, seek to improve women’s health outcomes by increasing women’s autonomy in health decision-making. The results of this study will also address the gap in knowledge in the existing research on the association of women’s health decision-making autonomy on their own healthcare and the country’s zones of residence. The study addressed the following questions: 1) To what extent and whether socio-demographic characteristics (age, education, residence, occupation, wealth index, and zones of the country) are independently associated with health decision-making autonomy? The study hypotheses were: 1) There is a significant association between sociodemographic factors and health decision-making autonomy. 2) There are regional/zone differences in Tanzanian women’s decision-making autonomy about their own healthcare.

## Materials and methods

### Design

This was a secondary data analysis of the Tanzania Demographic and Health Survey and Malaria Indicator Survey (TDHS-MIS) 2022. Data from the TDHS-MIS 2022 individual recode documentation were specifically used for this study. The TDHS-MIS is a nationwide survey conducted every five years. The original TDHS-MIS 2022 was undertaken by the Tanzanian National Bureau of Statistics (NBS) and the Office of the Chief Government Statistician Zanzibar (OCGS) in collaboration with affiliate governmental agencies, including the Ministries of Health (MoH) of Tanzania, both in the Mainland and Zanzibar, and the Tanzania Food and Nutrition Centre (TFNC) amongst other agencies. Technical assistance for the survey was provided by ICF International. The 2022 TDHS-MIS was undertaken using a multistage sampling design. The initial phase involved the selection of clusters (primary sampling units), which comprised enumeration areas defined during the 2012 Population and Housing Census. In all, 629 clusters were selected in phase 1, comprising 211 and 418 from urban and rural areas, respectively. The second phase involved systematically selecting households from the selected clusters during phase 1. Averagely, 26 households were selected from each cluster, making a projected total of 16,354 households for the survey. All women in their reproductive age (15–49 years) in selected households were eligible for participation, and those who consented were interviewed on a range of individual and population health issues, including themes on health decision-making autonomy.

### Characteristics of participants

All women of reproductive age from all regions in Tanzania. According to the World Health Organization (WHO), women of reproductive age are all women aged 15–49 years [34]. Study participants were selected to fit the eligibility criteria under the study: 1) women who were married or living with a partner at the time of the interview, 2) responded to the question, who usually decides on the respondent’s healthcare. A total of 9,249 women met these criteria.

### Study variables and measurements

#### Outcome variable.

The outcome variable for this study was women’s health decision-making autonomy. This variable was measured by using survey questionnaires in the TDHS-MIS data. This was obtained from the variable “decisions on personal health care.” Women were asked, “Who usually decides on the respondent’s healthcare?” Five responses were recorded, “respondent alone,” “respondent and partner,” “husband/partner alone,” “someone else,” and “other.” This was recoded at trilevel as “respondent alone =complete autonomy =1”, “respondent and partner =joint autonomy =2”, “partner alone =no autonomy =4”, and “someone else =no autonomy=5”. Respondents who answered “others” were dropped. Therefore, the three levels of autonomy considered in this study were “*No Autonomy,” “Joint Autonomy,”* and “*Complete Autonomy.* No Autonomy (partner alone and someone else) was selected as a baseline for interpretation, as it is considered the lowest category for women’s health decision-making autonomy [11]. Complete autonomy was considered a higher category for women’s decision-making autonomy. For this study, a woman’s autonomy was defined as the capacity of the woman to decide alone on their healthcare [12].

#### Independent variables.

The key explanatory variables for this study were the respondent’s sociodemographic characteristics. These variables were selected because our focus was to model specific attributes of women that correlate with their autonomy, as previous literature has shown the link between sociodemographic characteristics and women’s health decision-making autonomy [28,35]. Understanding how these variables interact can reveal a complex relationship surrounding women’s health decision-making autonomy. Therefore, the explanatory variables included were the woman’s age, level of education, working status, place of residence, household wealth index, and country’s zones. All the explanatory variables were categorical. The wealth index in the DHS data was a composite variable measure of a household’s cumulative living standard and relative wealth, calculated using data on the household’s ownership of selected assets, including types of water access and sanitation facilities, televisions and bicycles, and materials for housing construction among others assets. The resulting index was then presented in ordered categories: poorest, poorer, middle, richer, and richest. For statistical analysis, several variables were recoded into categories. The woman’s age was recoded into quinary age groups: 15–19 years, 20–24 years, 25–29 years, 30–34 years, 35–39 years, 40–44 years, and 45–49 years. Women aged 15–19 years were a reference group. The woman’s level of education was recoded as no education = 0, primary = 1, secondary = 2, higher = 3, whereas no education was a reference group: place of residence recoded as urban = 1, rural = 2; urban was a reference group: household wealth index as poorest = 1, poorer = 2, middle = 3, richer = 4, and richest = 5; whereas poorest was our reference group: currently working as currently working = 1, not currently working = 0; where our reference group was not currently working: and zones were recoded as Western = 1, Northen = 2, Central = 3, Southern Highlands = 4, Southern = 5, Southwest Highlands = 6, Lake = 7, Eastern = 8, and Zanzibar = 9: the Western zone was the reference group. The rationale for assigning a reference group for each variable is to ensure clarity of interpretation of the results and for clear comparison with the other categories [36].

### Statistical analysis

The R statistical programming language was used to perform the analysis. Data were imported into R programming language and cleaned using the appropriate packages in R. Descriptive statistics were calculated using frequencies. A chi-square was used to describe the sociodemographic characteristics of the respondents and their association with women’s health decision-making autonomy on their own health [Table 2]. A multivariable Ordinal Logistic Regression was used to assess the independent association between the explanatory and outcome variables. The variable selection in the regression model was determined by their significance associated with health decision-making autonomy at the bivariate analysis or as reported in the previous studies [26,27]. The Ordinal Logistic Regression was conducted using a single multivariable model. The coefficients were exponentiated to derive adjusted odds ratios (AORs) and corresponding 95% Confidence Intervals (CI) estimates. Statistically significant results were pegged at 0.05 alpha level. All univariate, bivariate, and multivariable analyses accounted for complex survey design.

### Ethical approval

The ethical approval for the DHS was the Institutional Review Board (IRB) of ICF International and the National Institute of Medical Research (NIMR), Tanzania. Field agents obtained informed consent from all eligible women prior to enrollment in the survey. The PI was granted permission to use the de-identified secondary data through online registration at the DHS website as https://www.dhsprogram.com/. No additional consent was required from participants in this secondary data analysis.

## Results

### Participants characteristics

A total sample size of 9,249 participants was included in this study. The participants were all married females aged 15–49 years drawn from 31 regions of Tanzania Mainland, and Zanzibar. A large proportion (20%) of women aged 25–29. The majority of women reside in rural areas, 69%. About 58% of women had attained a primary education. Most of the women came from the Lake zone (30%). It was observed that 63% of women were currently working and, 23% of women were from the richest households [Table 1].

**Table 1:** Sociodemographic characteristics of participants (N = 9249).

Variable	Frequence (n)	Percentage (%)
**Age -years**		
15–19	563	6.1
20–24	1,612	17.0
25–29	1,894	20.0
30–34	1,616	17.0
35–39	1,427	15.0
40–44	1,181	13.0
45–49	954	10.0
**Place of residence**		
Urban	2,894	31.0
Rural	6,355	69.0
**Education**		
No education	1,887	20.0
Primary	5,395	58.0
Secondary	1,861	20.0
Higher	106	1.1
**Wealth-index**		
Poorest	1,715	19.0
Poorer	1,713	19.0
Middle	1,760	19.0
Richer	1,970	21.0
Richest	2,090	23.0
**Currently-working**		
Not currently working	3,379	37.0
Currently working	5,869	63.0
**Zones**		
Western	806	8.7
Northen	1058	11.0
Central	948	10.0
Southern Highlands	541	5.8
Southern	453	4.9
Southwest Highlands	862	9.3
Lake	2774	30.0
Eastern	1519	16.0
Zanzibar	287	3.1
**Health decision making autonomy**		
No autonomy	2408	26.0
Joint autonomy	4933	53.0
Complete autonomy	1908	21.0

### Health decision-making autonomy about own healthcare

Only 21% of women reported being completely autonomous regarding decisions on their own health care, while the majority (53%) of women reported that health decisions about their own healthcare were made jointly, and 26% reported health decisions were made by husbands/partners alone and someone else.

### Factors associated with health decision-making autonomy on own healthcare

Cross-tabulation results show that socio-demographic characteristics are significantly associated with all three forms of women’s health decision-making autonomy. Women’s age, education level, place of residence, level of household wealth index, current working status, and country’s zones were statistically significantly associated with health decision-making autonomy [Table 2].

**Table 2:** Factors related to health decision-making autonomy on own healthcare (N = 9249).

Variable	No autonomy 2408 ^1^	Joint autonomy 4933 ^1^	Complete automomy 1908 ^1^	p-value^2^
**Age -years**				**<.001**
15–19	227 (9.4)	269 (5.5)	68 (3.5)	
20–24	532 (22)	803 (16)	277 (15)	
25–29	496 (21)	1,083 (22)	315 (17)	
30–34	382 (16)	896 (18)	337 (18)	
35–39	351 (15.)	718 (15)	357 (19)	
40–44	220 (9.1)	633 (13)	329 (17)	
45–49	199 (8.3)	530 (11)	224 (12)	
**Place of residence**				**<.001**
Urban	471 (20)	1,600 (32)	823 (43)	
Rural	1,937 (80)	3,333 (68)	1,084 (57)	
**Education**				**<.001**
No education	767 (32)	784 (16)	335 (18)	
Primary	1,341 (56)	2,959 (60)	1,095 (57)	
Secondary	297 (12)	1,121 (23)	442 (23)	
Higher	2 (<.1)	68 (1)	36 (2)	
**Wealth-index**				**<.001**
Poorest	717 (30)	740 (15)	258 (14)	
Poorer	534 (22)	873 (18)	307 (16)	
Middle	452 (19)	1,000 (20)	309 (16)	
Richer	396 (16)	1,139 (23)	435 (23)	
Richest	308 (13)	1,182 (24)	599 (31)	
**Currently-working**				**<.001**
Not currently working	1,182 (49)	1,620 (33)	578 (30)	
Currently working	1,226 (51)	3,313 (67)	1,330 (70)	
**Zones**				**<.001**
Western	484 (20)	217 (4.4)	105 (5.5)	
Northern	212 (8.8)	613 (12)	234 (12)	
Central	153 (6.4)	582 (12)	213 (11)	
Southern Highlands	50 (2.1)	423 (8.6)	68 (3.6)	
Southern	62 (2.6)	321 (6.5)	71 (3.7)	
Southwest Highlands	84 (3.5)	575 (12)	202 (11)	
Lake	892 (37)	1,318 (27)	563 (30)	
Eastern	407 (17)	739 (15)	373 (20)	
Zanzibar	64 (2.7)	144 (2.9)	79 (4.1)	

2 = chi-squared test with Rao and Scott’s second order of correction; ^1 ^= n (%).

### Multivariable ordinal logistic regression

Three categories were formed depending on how women’s autonomy in health decision-making is constructed in the literature [10,11]. In our analysis, we were interested in modeling the predictors for complete autonomy as a higher level of health decision-making autonomy among women. Therefore, the ordinal logistic regression was conducted to assess how the explanatory variables relate to different hierarchies of women’s health decision-making autonomy. Generally, the odds of having complete autonomy as opposed to no autonomy were greater with the increasing age of women. For example, the odds of women having complete health decision-making autonomy compared to no autonomy were two times greater (AOR = 2.15; 95% CI: 1.70, 2.71) for women 40–44 years old compared to those 15–19 years old, holding other variables constant. Likewise, the odds of having complete autonomy compared to no autonomy were greater with increasing levels of formal education relative to no formal education. For instance, the odds of women having complete autonomy as opposed to no autonomy were twice higher (AOR = 2.07; 95% CI: 1.39, 3.08) for women who had attained higher education than those with no formal education.

Similarly, the odds of having complete autonomy compared to no autonomy were greater with increasing levels of household wealth index. For instance, the results indicated that the odds of being completely autonomous as opposed to having no autonomy increased by 80% among women from the richest households relative to their counterparts from the poorest households (AOR = 1.80; 95% CI: 1.39, 2.33). The odds of having complete autonomy as opposed to no autonomy were 1.61 times higher (AOR = 1.61; 95% CI: 1.43, 1.83) for women who were currently working compared to those who were not currently working even after controlling for other variables. There were zonal variations in the autonomy status of women in Tanzania. Notably, the odds of having complete autonomy as opposed to no autonomy were approximately six times greater (AOR = 5.86; 95% CI: 4.47, 7.67) for women from the Southwest Highlands compared to the reference group (Western zone). On the contrary, women who resided in rural areas had 41% reduced odds of having complete autonomy compared to no autonomy than their urban counterparts (AOR = 0.59; 95% CI: 0.46, 0.75) [Table 3].

**Table 3:** Multivariate ordinal logistic regression on factors related to women’s health decision-making autonomy on their own healthcare.

Variable	OR	95% *CI*	AOR	95% *CI*
**Age -years**				
15–19	1		1	
20–24	1.42	[1.16, 1.74]	1.23	[1.00, 1.51]
25–29	1.73	[1.40, 2.13]	1.31	[1.06, 1.63]
30–34	2.08	[1.66, 2.60]	1.53	[1.23, 1.91]
35–39	2.28	[1.81 2.85]	1.79	[1.42, 2.26]
40–44	2.92	[2.34, 3.64]	2.15	[1.70, 2.71]
45–49	2.42	[1.92, 3.05]	1.86	[1.47, 2.35]
**Place of residence**				
Urban	1		1	
Rural	0.48	[0.41, 0.57]	0.59	[0.46, 0.75]
**Education**				
No education	1		1	
Primary	1.76	[1.48, 2.09]	1.36	[1.15, 1.60]
Secondary	2.47	[2.02, 3.04]	1.66	[1.34, 2.06]
Higher	4.58	[3.27, 6.39]	2.07	[1.39, 3.08]
**Wealth index**				
Poorest	1		1	
Poorer	1.52	[1.26, 1.83]	1.42	[1.20, 1.69]
Middle	1.78	[1.46, 2.17]	1.39	[1.16, 1.66]
Richer	2.37	[1.92, 2.92]	1.62	[1.33, 1.98]
Richest	3.32	[2.70, 4.09]	1.80	[1.39, 2.33]
**Currently-working**				
Not currently working	1		1	
Currently working	1.77	[1.56, 2.01]	1.61	[1.43, 1.83]
**Zones**				
Western	1		1	
Northern	4.85	[3.31, 7.11]	3.97	[3.00, 5.24]
Central	5.44	[3.75, 7.89]	4.92	[3.66, 6.62]
Southern Highlands	4.97	[3.73, 6.61]	3.41	[2.66, 4.37]
Southern	4.83	[3.55, 6.56]	4.07	[3.05, 5.42]
Southwest Highlands	6.58	[4.85, 8.92]	5.86	[4.47, 7.67]
Lake	3.20	[2.34, 4.37]	2.74	[2.14, 3.51]
Eastern	4.28	[3.04, 6.02]	2.27	[1.69, 3.06]
Zanzibar	5.33	[3.98, 7.15]	3.38	[2.58, 4.43]

OR = Unadjusted Odss Ratio; AOR = Adjusted Odds Ratio.

## Discussion

This study sought to determine whether women’s sociodemographic characteristics correlated with decision-making autonomy on their own health. The results show that women’s age, level of education, household wealth index, women currently working, and country’s zones independently correlated with health decision-making autonomy on their own health. The odds of being completely autonomous in health decision-making as opposed to no autonomy increased with age, formal education level, working status, and household wealth index. The country’s zones were also a determinant factor for women’s complete autonomy in health decision-making. Women living in rural areas had reduced odds of having complete autonomy in health decision-making on their own health compared to their counterparts. Furthermore, the findings showed only 21% of Tanzanian women were completely autonomous in health decision-making on own health. It was, however, noteworthy that the majority of the women’s health decision-making was done with husbands/partners (53%) and with someone else (24%).

A study conducted in Tanzania by Masawe et al. (2019) reported a 19% prevalence of health decision-making autonomy among married women. The current study established a 21% prevalence of complete health decision-making autonomy among women. This implied that the women in Tanzania still have limited complete autonomy in making health decisions on their own healthcare. This may be partly attributed to social and cultural norms, religious beliefs, and values influencing how women live and interact with their husbands/partners [2,37]. It can also be due to the social constructs and gender roles that many developing countries like Tanzania practice that restrict women’s full participation in decisions that affect their health [2,6]. A similar pattern was observed in Nigeria and Senegal, where only a small proportion of women, 6.2% and 6.26%, respectively, made health decisions in their healthcare [4,27]. A community-based cross-sectional study conducted in Ethiopia in 2021 reported a larger proportion (75.1%) of women’s health decision-making autonomy on their own health compared to our findings. The variations could possibly be explained by differences in data collection tools and procedures. The data collection tool in this study was developed by the researchers through reviewing various literature [25]. Additionally, this study was conducted in a specific area Debretabor, in northwest Ethiopia, whereas the results for our study are from the national demographic and health survey.

Women’s age was positively associated with complete autonomy in their own health decision-making. Similar findings have been reported in many studies using a national representative sample like ours in LMICs [22,38–40]. In Indonesia, women who were older than 35 years were reported to have higher decision-making autonomy in the household [40]. In Ethiopia, Nigatu et al. (2014) found that women aged 35–39 were approximately 4 times more likely to have higher autonomy regarding their own health than those aged <20. The possible reasons associated with this increase include that as women get older, their level of maturity increases, and therefore, they gain more independence in decisions and power [10,22].

This study also found a significant relationship between women’s level of education and complete autonomy in their own healthcare decisions. Specifically, it was observed that as the level of formal education increases, the chances of being completely autonomous in one’s health decisions as opposed to no autonomy increase. Higher-educated women were twice as likely to have complete autonomy than those without. This finding is consistent with other similar studies elsewhere [4,24,26,27,40], including one from Nigeria, where higher-educated women were 1.57 times more likely to make health decisions alone than those without [27]. Some possible reasons behind this finding are that educated women possess better knowledge about health issues, medical information, and healthcare options [41]. This awareness enables them to make informed choices regarding their health.

The household wealth index was directly associated with complete women’s health decision-making autonomy on their own health. Women from the richest households were 1.80 times more likely to make decisions alone than women from the poorest households. This result is consistent with studies done in Ethiopia [4,23,24,39]. It is also in accordance with a study conducted in Nigeria that found the richest women were 2.42 times more likely than the poorest to make health decisions alone [27]. In Ghana, it was reported that the wealth index influences maternal healthcare utilization [42,43].

Furthermore, this study found that women currently working had a direct association with complete autonomy in health decision-making on their own health compared to those not currently working. Women who were currently working were approximately twice as likely to have had complete autonomy as opposed to no autonomy compared to those not currently working. Our findings are similar to studies conducted elsewhere in Ethiopia and Nepal [22,23,39]. Alemayehu and Meskele (2017) found that merchant women from rural districts of Southern Ethiopia were almost two times more likely than housewives to have their healthcare decision-making autonomy. This suggests that both economic engagement and the nature of one’s work can play a critical role in empowering women regarding their health choices [44].

The strongest magnitude of association observed with women’s decision-making autonomy was the country zones. As we hypothesized, country zones were an important determinant for Tanzanian women’s decision-making autonomy, even after controlling for other factors. Tanzania is divided into nine zones (Western, Northern, Central, Southern Highlands, Southern, Southwest Highlands, Lake, Eastern, and Zanzibar). The Southwest Highlands zones encompass several regions (Mbeya, Rukwa, Katavi, Songwe) with diverse sociodemographic characteristics [12]. The area is home to various ethnic groups, including the Bantu and others, contributing to a rich cultural mosaic. A significant portion of the population is young, and agriculture is the primary economic activity, which together creates both opportunities and challenges for the region [12].

Women living in the Southwest Highlands were five times more likely to decide alone on matters affecting their health compared with the Western zones. This study lays the groundwork for this particular finding. Masawe et al. (2019) did not assess the country’s zone as a determinant for women’s health decision-making autonomy in their own healthcare. Other studies in African countries [4,24,27,39] have also found that geographical locations were independently associated with women’s autonomy. These findings imply that there are factors associated with geographical locations that are yet to be explicated. A study in India reported that women in the Southern regions of India have more exposure to the outside world, a greater voice in family life, and more freedom of movement than those in the north [45,46]. Osamor and Grady (2018) found that Nigerian women from the Southwest region were 8.3 times more likely to make their own healthcare decisions than women from the Northwest region. Very little is known about the influence of zones and women’s health decisions in Tanzania. But family life in Tanzania, like most parts of African countries, is guided by descriptive norms and belief systems that derive from culture and ethnicity. Cultural norms, social norms, and beliefs are central components of regional variations that may partly explain the independent influence of region on women’s autonomy.

Women who resided in rural areas were significantly less likely to make their own health decisions than those from urban areas. These findings were consistent with others conducted in LMICs [10,22,26,27]. The role of place of residence in decision-making has now been documented beyond doubt in affecting the health of individuals living there. In Tanzania, about (66%) of the population live in rural areas [12]. The geographic isolation of rural populations and the challenges they face in meeting basic social services, economic opportunities, and healthcare services are profound.

### Strengths and limitations

This study has several strengths and weaknesses. One key strength is the representative nature of the data among the Tanzanian women population. To the author’s best knowledge, this is the first study in Tanzania using nationally representative data to establish the findings on the country’s zone as the strongest determinant of women’s health decision-making autonomy in their own healthcare. The use of a large nationally representative dataset (TDHS-MIS 2022–2023) enhances the reliability and generalizability of the findings, allowing for a more accurate reflection of the diverse sociodemographic factors influencing health decision-making autonomy across different populations.

The study had the following limitations. Because this was a cross-sectional study, a causal relationship between sociodemographic characteristics (explanatory variables) and health decision-making autonomy (outcome variable) can not be established. Therefore, the conclusion of this paper was based on the association relationship between the explanatory and outcome variables. Since this study was a secondary data analysis, the responses were subjected to recall biases. Also, the nature of the survey questions (closed-ended) has limited the ability to capture the context and nuance of the responses. For example, in this study, we interpreted health decisions made by a woman alone as the higher hierarchy representing women’s autonomy, which could also indicate a lack of support from the husband/partner. To assert this interpretation appropriately, follow-up qualitative prompts are needed to clarify this ambiguity. The study measures autonomy based on the ability of women to make health-related decisions, which might not adequately represent the complexity of household decision-making processes.

### Implications to practice, policy, and future research

The study underscores the urgent need for a qualitative study to gain deeper insights into women’s experiences regarding their healthcare decision-making autonomy. While this study highlighted the associations between health decision-making autonomy on their own health and sociodemographic factors, qualitative research can explore the nuanced barriers and facilitators women face in navigating the process of health decision-making autonomy. Additionally, since the results are based on an association relationship, future research can utilize longitudinal designs to better understand the causal relationships between the identified factors and women’s health decision-making autonomy over time. Stakeholders can create focused interventions to improve women’s health decision autonomy, emphasizing education and initiatives that generate income, especially in rural regions. Policymakers are encouraged to continue creating policies that promote women’s education and economic empowerment, as these factors are linked to increased autonomy in healthcare decisions.

## Conclusion

The results show that health decision-making autonomy on healthcare among Tanzanian women remains very low. More than half of married women in Tanzania reported having no capacity to decide on matters related to their own healthcare. Factors associated with women’s health decision-making autonomy include women’s age, level of education, place of residence, household wealth index, women currently working, and the country’s zones. Interventions and policies targeting modifiable factors such as education and income-generating activities that increase women’s decision-making autonomy will improve their health outcomes and that of the family and community. Further qualitative research is needed to explore how women interact and navigate various components of health decisions (maternal health, clinic visits, blood transfusion, etc).

### Recommendations

Accelerated and concerted efforts to increase health decision-making autonomy among married women will eventually improve their health and well-being and that of society. The significant zonal variations in autonomy observed in the study call for further investigation. Understanding the fundamental causes of these disparities could help develop more region-specific and successful strategies.

## References

[1] Budu E, Seidu A-A, Armah-Ansah EK, Sambah F, Baatiema L, Ahinkorah BO. Women’s autonomy in healthcare decision-making and healthcare seeking behaviour for childhood illness in Ghana: Analysis of data from the 2014 Ghana Demographic and Health Survey. PLoS One. 2020;15(11):e0241488. doi: 10.1371/journal.pone.0241488 33166370 PMC7652316

[2] Garrison-Desany HM, Wilson E, Munos M, Sawadogo-Lewis T, Maïga A, Ako O, et al. The role of gender power relations on women’s health outcomes: evidence from a maternal health coverage survey in Simiyu region, Tanzania. BMC Public Health. 2021;21(1):909. doi: 10.1186/s12889-021-10972-w 33980197 PMC8117490

[3] Sialubanje C, Massar K, Hamer DH, Ruiter RAC. Reasons for home delivery and use of traditional birth attendants in rural Zambia: a qualitative study. BMC Pregnancy Childbirth. 2015;15:216. doi: 10.1186/s12884-015-0652-7 26361976 PMC4567794

[4] Sougou NM, Bassoum O, Faye A, Leye MMM. Women’s autonomy in health decision-making and its effect on access to family planning services in Senegal in 2017: a propensity score analysis. BMC Public Health. 2020;20(1):872. doi: 10.1186/s12889-020-09003-x 32503492 PMC7275346

[5] Seidu A-A, Aboagye RG, Okyere J, Agbemavi W, Akpeke M, Budu E, et al. Women’s autonomy in household decision-making and safer sex negotiation in sub-Saharan Africa: An analysis of data from 27 Demographic and Health Surveys. SSM Popul Health. 2021;14:100773. doi: 10.1016/j.ssmph.2021.100773 33855160 PMC8025044

[6] Woldemicael G, Tenkorang EY. Women’s autonomy and maternal health-seeking behavior in Ethiopia. Matern Child Health J. 2010;14(6):988–98. doi: 10.1007/s10995-009-0535-5 19882240

[7] WHO. SDGs Target 3.1. Maternal mortality by 2030: Fact sheet. World Health Organization; 2023. Available: https://www.who.int/data/gho/data/themes/topics/sdg-target-3-1-maternal-mortality

[8] WHO. Maternal mortality fact sheet. In: World Health Organization; 2014. doi:https://apps.who.int/iris/bitstream/handle/10665/112318/WHO_RHR_14.06_eng.pdf

[9] Bloom SS, Wypij D, Das Gupta M. Dimensions of women’s autonomy and the influence on maternal health care utilization in a north Indian city. Demography. 2001;38(1):67–78. doi: 10.1353/dem.2001.0001 11227846

[10] Nigatu D, Gebremariam A, Abera M, Setegn T, Deribe K. Factors associated with women’s autonomy regarding maternal and child health care utilization in Bale Zone: a community based cross-sectional study. BMC Womens Health. 2014;14:79. doi: 10.1186/1472-6874-14-79 24990689 PMC4094397

[11] Osamor PE, Grady C. Women’s autonomy in health care decision-making in developing countries: a synthesis of the literature. Int J Womens Health. 2016;8:191–202. doi: 10.2147/IJWH.S105483 27354830 PMC4908934

[12] Ministry of Health (MoH) TM, Ministry of Health (MoH) Z, National Bureau of Statistics N, Office of the Chief Government Statistician O, ICF. Demographic and Health Survey and Malaria Indicator Survey 2022 Final Report. Dodoma, Tanzania, and Rockville, Maryland, USA; 2022.

[13] Langer A, Meleis A, Knaul FM, Atun R, Aran M, Arreola-Ornelas H, et al. Women and Health: the key for sustainable development. Lancet. 2015;386(9999):1165–210. doi: 10.1016/S0140-6736(15)60497-4 26051370

[14] Pradhan MR, Unisa S, Rawat R, Surabhi S, Saraswat A, R S R, et al. Women empowerment through involvement in community-based health and nutrition interventions: Evidence from a qualitative study in India. PLoS One. 2023;18(4):e0284521. doi: 10.1371/journal.pone.0284521 37079532 PMC10118087

[15] Kassahun A, Zewdie A. Decision-making autonomy in maternal health service use and associated factors among women in Mettu District, Southwest Ethiopia: a community-based cross-sectional study. BMJ Open. 2022;12(5):e059307. doi: 10.1136/bmjopen-2021-059307 35501088 PMC9062806

[16] Mondal D, Karmakar S, Banerjee A. Women’s autonomy and utilization of maternal healthcare in India: Evidence from a recent national survey. PLoS One. 2020;15(12):e0243553. doi: 10.1371/journal.pone.0243553 33296428 PMC7725295

[17] Olwanda E, Opondo K, Oluoch D, Croke K, Maluni J, Jepkosgei J, et al. Women’s autonomy and maternal health decision making in Kenya: implications for service delivery reform - a qualitative study. BMC Womens Health. 2024;24(1):181. doi: 10.1186/s12905-024-02965-9 38504293 PMC10949706

[18] Thaddeus S, Maine D. Too far to walk: maternal mortality in context. Soc Sci Med. 1994;38:1091–110. 8042057 10.1016/0277-9536(94)90226-7

[19] Charlet D, Moran AC, Madhavan S. Summary findings from a mixed methods study on identifying and responding to maternal and newborn illness in seven countries: implications for programs. J Health Popul Nutr. 2017;36(Suppl 1):48. doi: 10.1186/s41043-017-0126-9 29297392 PMC5764052

[20] Wallace HJ, McDonald S, Belton S, Miranda AI, da Costa E, Matos L da C, et al. The decision to seek care antenatally and during labour and birth - Who and what influences this in Timor-Leste? A qualitative project exploring the perceptions of Timorese women and men. Midwifery. 2018;65:35–42. doi: 10.1016/j.midw.2018.05.013 30055403

[21] D’Ambruoso L, Byass P, Qomariyah SN, Ouédraogo M. A lost cause? Extending verbal autopsy to investigate biomedical and socio-cultural causes of maternal death in Burkina Faso and Indonesia. Soc Sci Med. 2010;71(10):1728–38. doi: 10.1016/j.socscimed.2010.05.023 20646807

[22] Acharya DR, Bell JS, Simkhada P, van Teijlingen ER, Regmi PR. Women’s autonomy in household decision-making: a demographic study in Nepal. Reprod Health. 2010;7:15. doi: 10.1186/1742-4755-7-15 20630107 PMC2914657

[23] Alemayehu M, Meskele M. Health care decision making autonomy of women from rural districts of Southern Ethiopia: a community based cross-sectional study. Int J Womens Health. 2017;9:213–21. doi: 10.2147/IJWH.S131139 28458582 PMC5402916

[24] Asabu MD, Altaseb DK. The trends of women’s autonomy in health care decision making and associated factors in Ethiopia: evidence from 2005, 2011 and 2016 DHS data. BMC Womens Health. 2021;21(1):371. doi: 10.1186/s12905-021-01517-9 34702222 PMC8547022

[25] Kebede AA, Cherkos EA, Taye EB, Eriku GA, Taye BT, Chanie WF. Married women’s decision-making autonomy in the household and maternal and neonatal healthcare utilization and associated factors in Debretabor, northwest Ethiopia. PLoS One. 2021;16(9):e0255021. doi: 10.1371/journal.pone.0255021 34570781 PMC8476028

[26] Masawe C, Mcharo E, Masawe G. Women’autonomy in decision-making on own healthcare in tanzania. J Educ Humanit Sci. 2019;8.

[27] Osamor P, Grady C. Factors associated with women’s health care decision-making autonomy: empirical evidence from Nigeria. J Biosoc Sci. 2018;50(1):70–85. doi: 10.1017/S0021932017000037 28183370 PMC6010313

[28] Gebeyehu NA, Gelaw KA, Lake EA, Adela GA, Tegegne KD, Shewangashaw NE. Women decision-making autonomy on maternal health service and associated factors in low- and middle-income countries: Systematic review and meta-analysis. Womens Health (Lond). 2022;18:17455057221122618. doi: 10.1177/17455057221122618 36062751 PMC9445465

[29] Yuill C, McCourt C, Cheyne H, Leister N. Women’s experiences of decision-making and informed choice about pregnancy and birth care: a systematic review and meta-synthesis of qualitative research. BMC Pregnancy Childbirth. 2020;20(1):343. doi: 10.1186/s12884-020-03023-6 32517734 PMC7285707

[30] Shanto HH, Al-Zubayer MA, Ahammed B, Sarder MA, Keramat SA, Hashmi R, et al. Maternal Healthcare Services Utilisation and Its Associated Risk Factors: A Pooled Study of 37 Low- and Middle-Income Countries. Int J Public Health. 2023;68:1606288. doi: 10.3389/ijph.2023.1606288 37936874 PMC10625904

[31] Batura N, Poupakis S, Das S, Bapat U, Alcock G, Skordis J, et al. Factors associated with women’s healthcare decision-making during and after pregnancy in urban slums in Mumbai, India: a cross-sectional analysis. BMC Public Health. 2022;22(1):743. doi: 10.1186/s12889-022-13216-7 35418068 PMC9009007

[32] USAID. Gender equality and women’s empowerment. 2023 [cited 4 Mar 2024]. Available: https://www.usaid.gov/tanzania/gender-equality-and-womens-empowerment

[33] UNDP. UNDP Tanzania gender equality strategy 2022-2027. Dar es Salaam, Tanzania; 2022.

[34] WHO. The global health observatory: Explore a world of health data. 2019. Available: https://www.who.int/data/gho/indicator-metadata-registry/imr-details/women-of-reproductive-age-(15-49-years)-population-(thousands)

[35] Idris IB, Hamis AA, Bukhori ABM, Hoong DCC, Yusop H, Shaharuddin MA-A, et al. Women’s autonomy in healthcare decision making: a systematic review. BMC Womens Health. 2023;23(1):643. doi: 10.1186/s12905-023-02792-4 38042837 PMC10693143

[36] Learn statistics easily. Learn data analysis now. 28 Mar 2024 [cited 9 Oct 2024]. Available: https://statisticseasily.com/

[37] Yeates K, Chard S, Eberle A, Lucchese A, West N, Chelva M. She needs permission: A qualitative study to examine barriers and enablers to accessing maternal and reproductive health services among women and their communities in rural Tanzania. Afr J Reprod Health. 2021;25:139–52. doi: 10.29063/ajrh2021/v25i3s.12

[38] Adhikari R. Effect of Women’s autonomy on maternal health service utilization in Nepal: a cross sectional study. BMC Womens Health. 2016;16:26. doi: 10.1186/s12905-016-0305-7 27177683 PMC4867085

[39] Mare KU, Aychiluhm SB, Tadesse AW, Abdu M. Married women’s decision-making autonomy on contraceptive use and its associated factors in Ethiopia: A multilevel analysis of 2016 demographic and health survey. SAGE Open Med. 2022;10:20503121211068719. doi: 10.1177/20503121211068719 35083044 PMC8785292

[40] Rizkianti A, Afifah T, Saptarini I, Rakhmadi MF. Women’s decision-making autonomy in the household and the use of maternal health services: An Indonesian case study. Midwifery. 2020;90:102816. doi: 10.1016/j.midw.2020.102816 32823256

[41] Gumà J, Solé-Auró A, Arpino B. Examining social determinants of health: the role of education, household arrangements and country groups by gender. BMC Public Health. 2019;19(1):699. doi: 10.1186/s12889-019-7054-0 31170953 PMC6555096

[42] Ameyaw EK, Tanle A, Kissah-Korsah K, Amo-Adjei J. Women’s Health Decision-Making Autonomy and Skilled Birth Attendance in Ghana. Int J Reprod Med. 2016;2016:6569514. doi: 10.1155/2016/6569514 28116348 PMC5220507

[43] Amugsi DA, Lartey A, Kimani E, Mberu BU. Women’s participation in household decision-making and higher dietary diversity: findings from nationally representative data from Ghana. J Health Popul Nutr. 2016;35(1):16. doi: 10.1186/s41043-016-0053-1 27245827 PMC5026004

[44] Onarheim KH, Iversen JH, Bloom DE. Economic Benefits of Investing in Women’s Health: A Systematic Review. PLoS One. 2016;11(3):e0150120. doi: 10.1371/journal.pone.0150120 27028199 PMC4814064

[45] BasuMalwade A. Culture, the status of women, and demographic behaviour: illustrated with the case of India. Clarendon Press; 1992.

[46] Jejeebhoy SJ, Sathar ZA. Women’s Autonomy in India and Pakistan: The Influence of Religion and Region. Population & Development Rev. 2001;27(4):687–712. doi: 10.1111/j.1728-4457.2001.00687.x

